# Endurance of the Dorsal and Ventral Muscles in the Neck

**DOI:** 10.3390/jfmk5030047

**Published:** 2020-07-08

**Authors:** Cameron M. Liss, Adeola A. Sanni, Kevin K. McCully

**Affiliations:** Department of Kinesiology, University of Georgia, Athens, GA 30602, USA; cameron.liss25@uga.edu (C.M.L.); mccully@uga.edu (K.K.M.)

**Keywords:** neck muscles, muscle fatigue, fatigability, human subjects, myography, neuromuscular electrical stimulation

## Abstract

Endurance of the muscles of the neck are rarely studied. This study measured the endurance index (EI) of the sternocleidomastoid (SCM) and upper trapezius muscles of the neck (trap). The vastus lateralis (VL) was used for comparison. Skeletal muscle endurance of twelve healthy subjects, age 19–22 years, were tested on their SCM and trap in random order on one day, VL was tested on a separate day. Participants were tested in the supine position for the SCM and VL muscles and the prone position for the trap. Muscle contractions consisted of a 5 Hz twitch electrical stimulation for 5 min. Muscle acceleration (resultant vector) was measured using a triaxial accelerometer. EI was the ending acceleration as a percentage of the maximal acceleration. The endurance index (EI) for the SCM, trap, and VL was 42.3 ± 13.0%, 42.3 ± 20.2%, and 92.9 ± 11.0%, respectively. The EI of the VL was significantly higher than the EI of the SCM (*t*(2,22) = 10.33, *p* < 0.001) and the trap (*t*(2,22) = 7.625, *p* < 0.001). The EI was not different between the SCM and the trap muscle (*t*(2,22) = 0.004, *p* = 0.997). In conclusion, the neck muscles had much less endurance than the muscles in the leg and could make fatigued athletes more susceptible to concussions caused by head impacts.

## 1. Introduction

Muscles in the neck play a vital role in maintaining the position and movement of the head. The sternocleidomastoid (SCM) and the trapezius (trap) are the largest muscles in the neck [1]. The SCM can function unilaterally such as in lateral flexion and head rotation, and bilaterally such as in head elevation. The SCM originates from the manubrium and the clavicle and inserts majorly onto the mastoid process behind the ear [1]. The trap is a larger muscle with various origins and insertions; it is a wide and flat muscle that spreads across the upper neck and the back. The trap helps in twisting and turning the neck. The trap also controls the abduction and adduction of the head. The trap muscle extends from the occipital bone to the thoracic membrane and reaches the side onto the spine of the scapula.

The SCM and trapezius muscles stabilize and support the head and may influence injury risk [2]. Various sports and games such as football, basketball, rugby, hockey, etc. require load and impact on the neck muscles during play. Stronger neck muscles help brace for support and reduce an athlete’s risk for concussion or neck injury during a collision [3]; it is also believed that stronger neck muscles help improve head kinematic response after an impact [4]. Studies have also shown an increase in neck muscle pain among adolescents due to sitting position [5], the use of computers, and reduce muscle endurance [6]. Oliveira et al. reported a strong relationship between neck muscle pain and neck muscle endurance [7].

Several different techniques have been used to evaluate the strength and endurance of the neck muscles using voluntary contraction/isometric tests [8,9]. However, the use of maximal voluntary efforts in these tests requires the subjects to be highly motivated and familiar with the test protocol. A recent study evaluated the endurance of the neck muscles using involuntary twitch contractions produced with electrical stimulation [10], this study found that the lower trapezius muscle in the shoulder region had reduced muscle endurance compared to muscles in the arms and legs. However, similar measurements have not been performed on muscles in the neck.

Assessment of muscle endurance using involuntary twitch contractions involves measuring changes in muscle acceleration of the twitch contractions over a given time. This approach has been used to characterize muscle endurance in various human muscles [11,12,13]. The present study evaluated specific muscle endurance using twitch contractions and accelerometry in two muscles located in the neck; the SCM and the trap muscles, and compared them to the vastus lateralis (VL) muscle in the leg.

## 2. Materials and Methods

### 2.1. Participants

Twelve healthy participants (6 females, 6 males) between ages 19 to 22 years were recruited to participate in this study. All the subjects reported being physically active, however, none of the subjects performed regular exercise involving the neck muscles. Each participant was tested on two separate days. The study was approved by the University of Georgia Institutional Review Board, study00004412, approved on December 2, 2017, until December 1, 2020. All subjects provided written, informed consent before any testing. The demographic characteristics of the subjects are shown in Table 1.

### 2.2. Experimental Protocol

A within-subject experimental design was used for this study and a convenience sampling method was adopted to recruit the participants tested. The study was performed using the STROBE guidelines. Subjects were tested on two separate days. On the first day, their SCM and trap muscles were tested in alternating order. The participants laid in a supine position for testing of the SCM and a prone position for testing of the trap. The right and left muscles were randomly selected. On the second day, the VL was tested in the supine position, the right and left legs were also randomly selected.

### 2.3. Endurance Test

**Figure 1:**
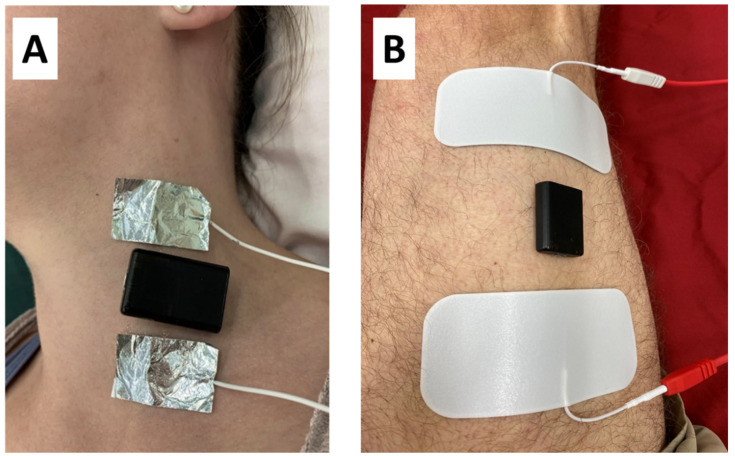
(**a**) The placement of the foil electrodes placed on the SCM (sternocleidomastoid) muscle, along with the tri-axial accelerometer (black). (**b**) The placement of the gel electrodes and accelerometer on the VL (vastus lateralis) muscle along with the tri-axial accelerometer (black).

Muscle endurance was measured using a 5 min stimulation protocol, similar to a previously published nine-minute endurance protocol [10,11,12,14,15]. Custom-made foil electrodes were placed on the neck and ultrasound gel was placed underneath the electrodes. The electrodes were cut to fit the approximate width of the muscles being tested. The electrodes were additionally held in place with surgical tape. A tri-axial accelerometer (Axivity-AX3, Newcastle Upon Tyne, UK) was placed in between the two foil electrodes with double-sided tape. The accelerometer was set at ±2 g, with 13-bit resolution, and 400 Hz sampling rate. For the VL testing, two gel electrodes were placed across the VL and an accelerometer was placed in between the two electrodes with double-sided tape (Figure 1).

The muscle was stimulated with a submaximal current level that shows a good visual contraction but comfortable for the participant. The stimulation intensity was adjusted to get a vigorous contraction and varied among participants. Stimulation current level ranged between 25 mA and 30 mA for the SCM and trap, and between 35 mA and 45 mA for the VL. Electrical stimulation produced a pulse interval of 200 µs/50 µs. The endurance test protocol used in this study consisted of 5 min of electrical stimulation at 5 Hz. The endurance index (EI) was calculated by dividing a few twitches at the end of the acceleration by the peak twitches at the start and multiplying by one hundred.

### 2.4. Adipose Tissue Thickness (ATT)

Subcutaneous ATT (adipose tissue thickness) was assessed using ultrasound B-mode imaging (LOGIQe; GE Healthcare, Chicago, IL, USA) with musculoskeletal scanning pre-set and a multifrequency linear transducer (8–12 MHz). The placement of the accelerometer on the neck and quadriceps was marked and then a static ultrasound image was taken.

### 2.5. Data Analysis

**Figure 2:**
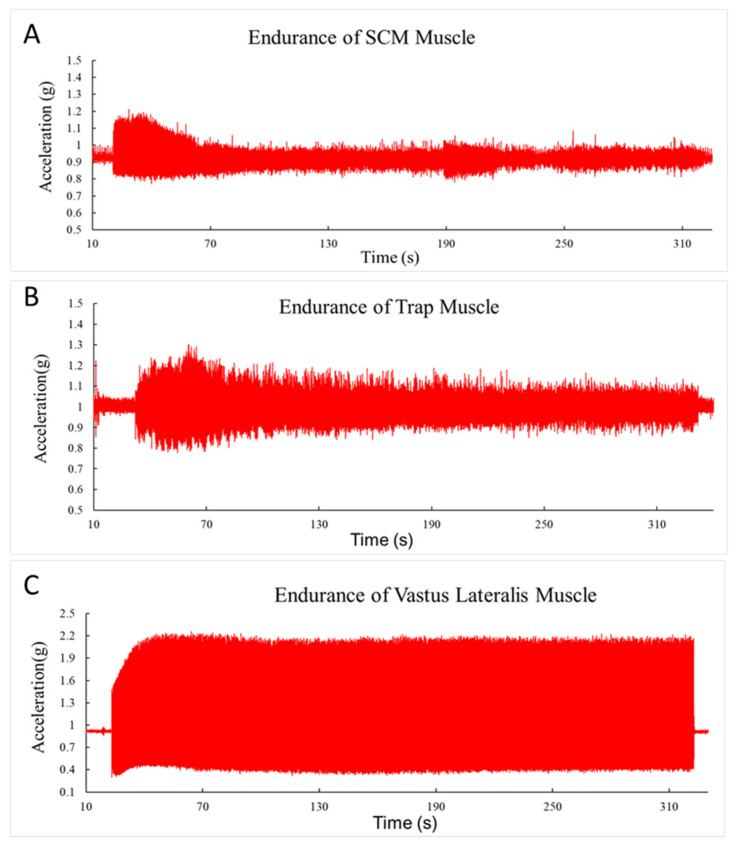
(**a**) Representative examples of 5-min endurance tests for the sternocleidomastoid muscles at 5 Hz. The decrease in acceleration overtime is calculated as the endurance index (EI). The y-axis is the acceleration (g) and the x-axis is time (secs). (**b**) Representative examples of 5-min endurance tests for the trap muscles at 5 Hz. The decrease in acceleration overtime is calculated as the endurance index (EI). The y-axis is the acceleration (g). (**c**) Representative examples of 5-min endurance tests for the vastus lateralis muscles at 5 Hz. The decrease in acceleration overtime is calculated as the endurance index (EI). The y-axis is the acceleration (g).

Data from the accelerometer were transferred to Microsoft Excel and a resultant vector was calculated from the three axes Ar = √(X² + Y² + Z²). Further analysis was done in MATLAB R2017b (Mathworks inc., Natick, MA, USA) using a customized written program. Peak-to-peak analysis was employed to determine the magnitude of acceleration for each contraction frequency. Endurance index was calculated as the percent of acceleration at the end of each stimulation frequency in relation to its peak value. Figure 2 shows examples of the acceleration during the endurance test for each muscle.

### 2.6. Statistical Analysis

Statistical analysis was done using SPSS version 24. Three-way analysis of variance (ANOVA) was used to find the difference in EI among the three muscles. Further post hoc test was done using an independent t-test to find the difference between the EI of the VL and the SCM, VL and trap, and SCM and trap, respectively. Significance was accepted at alpha level ≤0.05. ANOVA was also used to find the difference in ATT among the muscle, with a further post hoc analysis carried out to find the difference between the two muscles, respectively. A sample size of 12 was chosen based on a minimum detectable difference for endurance index values of 10%. It was estimated that a 10% minimum detectable difference could be achieved with a sample size of 12, assuming type 1 error levels of 5% and power of 80%, and a standard deviation of 12% of the mean.

## 3. Results

The ATT over the SCM was 0.3 ± 0.1 cm and the ATT over the trap was 0.4 ± 0.2 while the ATT for the VL was 1.0 ± 0.4 cm. A Shapiro–Wilk’s test and a visual inspection of their histogram, Q–Q plots and box plots show that the ATT scores were normally distributed for the SCM (*p* = 0.398) and the VL (*p* = 0.713) but not normally distributed for the trap (0.002). A Kruskal–Wallis non-parametric ANOVA test showed that there was a significant difference among the ATT over the three muscles (*p* < 0.001), with a further pairwise post hoc analysis showing there was no significant difference between the ATT over the SCM muscle and the trap muscle (*p* = 0.31). There was a positive significant correlation between ATT and EI of the muscle (*r* = 682, *p* < 0.0001).

**Figure 3:**
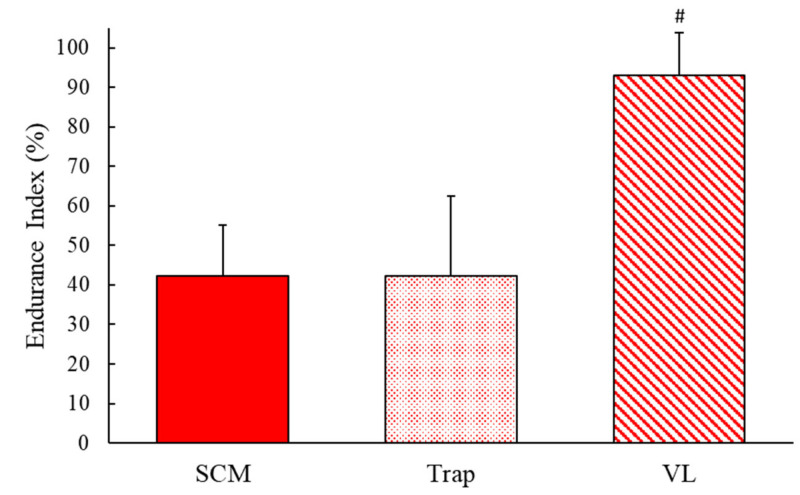
The endurance index mean and standard deviation of the sternocleidomastoid, trapezius, and vastus lateralis muscles. # shows a significant difference from both SCM and trap, *p* < 0.05.

The EI for the SCM, trap and VL was 42.3 ± 13.0%, 42.3 ± 20.2%, and 92.9 ± 11.0%, respectively (Figure 3). One-way ANOVA showed a significant difference between the muscles (F(2,22) = 57.4, *p* < 0.001), and a further post hoc analysis showed that the EI of the SCM was significantly lower than the EI of the VL (*t*(2,22) = 10.33, *p* < 0.001). The EI of the trap was also significantly lower than the VL (*t* (2,22) = 7.625, *p* < 0.001). There was no significant difference between the SCM and the trap muscles (*t* (2,22) = 0.004, *p* = 0.997). A one-way ANOVA showed homogeneity of variance (*p* = 0.20).

## 4. Discussion

This study showed the endurance of two muscles of the neck (sternocleidomastoid and trapezius) is considerably lower than the endurance of a muscle of the thigh. This result is similar to that reported for the lower trapezius [10]. In that study, the endurance index after a nine-minute endurance protocol was around 43% at 6 Hz stimulation compared to 42% for 5 Hz stimulation for the SCM and upper trap muscles in this study. In both that study and this study, the leg muscles showed very little fatigue as a result of 5 or 6 Hz electrical stimulation [15]. Other studies have reported the results of endurance tests for the neck muscles [16]. Differences in methodologies make comparisons difficult, as these studies used a test of task failure (duration of holding a weight). These results do suggest that neck muscles have reduced muscle endurance compared to limb muscles.

There have been a variety of approaches used to study muscle fatigue [17,18,19,20,21]. This study used a triaxial accelerometer to measure changes in muscle contractile speeds as an indication of muscle fatigue. Previous studies have used accelerometry to study muscle contractile function [22,23]. In addition, an accelerometer has been used to evaluate muscle endurance as changes in twitch contraction speeds [10,11,12,14,15]. These studies have shown a good relationship between muscle accelerometry and muscle mitochondrial capacity in clinical populations [14]. Other approaches to measure muscle contractile function have used task failure in response to an applied load [16,18]. Previous studies have also used electromyography (EMG) to measure changes associated with fatigue [16,24], even though there are limitations to using EMG signals to represent muscle fatigue [25,26]. Overall, using twitch accelerometry to study muscle endurance in the neck muscles has the advantage of not requiring strenuous voluntary efforts that are either difficult or potentially harmful.

A number of studies have evaluated the potential impact of muscle fatigue or reduced muscle function in the neck. Studies have shown an increase in SCM fatigability among patients with neck pain compared to a control group using a surface electromyography endurance test [16]. Jull et al. [27] reported that lower muscle endurance shows increase in persistent neck pain. Furthermore, Elham et al. [28] investigated neck pain among elementary students that showed high prevalence of neck pain due to sitting posture and use of backpacks. Studies have also reported reduced muscle function in the neck muscles of patients who report chronic pain symptoms or spinal abnormalities [6,16]. In our study, we only evaluated healthy young adults. Although it is not clear if low neck muscle endurance could be clinically harmful in healthy young adults, neck muscle strength has been associated with concussion risk in soccer players. It is possible that low neck muscle endurance could lead to neck fatigue during soccer matches, and thus increase concussion risk. Future studies are needed to evaluate the potential relationship between neck muscle endurance and injury risk in competitive athletics.

There are some limitations to our study. We did not evaluate participants who might be expected to have high muscle endurance in the neck muscles, as participants were generally healthy college students. Future studies might test subjects with endurance-trained neck muscles. Also, we did not compare our measurements with alternative measurements of muscle endurance in the neck. However, neck muscle endurance in previous studies using different methods reported results similar to our study [9]. Previous approaches to measuring endurance in neck muscles require voluntary effort and are more subjective than the endurance index in this study. A potential limitation of our study was the use of submaximal electrical stimulation. Although the advantage of using submaximal stimulation is that the lower levels of stimulation are more tolerable to the participants, this does not cause large movements of the head which might complicate the acceleration measurements. Previous studies have suggested that the endurance index is not influenced by submaximal stimulation levels [11]. This study shows there was a relationship between the adipose tissue over the muscle tested and the muscle endurance and thus future studies might look at the effect of fat on muscle endurance.

## 5. Conclusions

The supporting muscles of the neck have lower muscle endurance (more fatigable) than leg muscles in healthy young subjects. Twitch accelerometry has the potential to noninvasively evaluate muscle function in the neck, in a way comparable to tests of other muscles in the body. Future studies might evaluate neck muscle endurance and the potential impact of endurance training on athletes with the risk of concussion, or patients with chronic neck pain.

## Figures and Tables

**Table 1:** Characteristics of the participants.

Gender	Age Years	Height M	Weight Kg	BMI Kg/M^2^	SCM Right - Left	Trap Right - Left	VL Right - Left
Male	6	1.82 (1.12)	78.9 (14.1)	23.7 (1.7)	4R - 2L	2R - 4L	4R - 2L
Female	6	1.60 (0.06)	64.7 (9.6)	25.3 (2.6)	1R - 5L	5R - 1L	4R - 2L

SCM is sternocleidomastoid, trap is upper trapezius muscles, VL is vastus lateralis muscle. Values are means (SD). Each muscle number represents the distribution of muscle testing between the right and the left muscles.
